# Pyrolysis Kinetic Behavior and Thermodynamic Analysis of PET Nonwoven Fabric

**DOI:** 10.3390/ma16186079

**Published:** 2023-09-05

**Authors:** Samy Yousef, Justas Eimontas, Nerijus Striūgas, Alaa Mohamed, Mohammed Ali Abdelnaby

**Affiliations:** 1 Department of Production Engineering, Faculty of Mechanical Engineering and Design, Kaunas University of Technology, LT-51424 Kaunas, Lithuania; 2 Laboratory of Combustion Processes, Lithuanian Energy Institute, Breslaujos 3, LT-44403 Kaunas, Lithuania; 3 Department of Production Engineering and Printing Technology, Akhbar Elyom Academy, 6th of October 12566, Egypt; alakha@kth.se; 4 Mechatronics Systems Engineering Department, October University for Modern Sciences and Arts-MSA, Giza 12451, Egypt

**Keywords:** PET nonwoven fabric, pyrolysis, TG/FTIR-GC/MS analysis, pyrolysis kinetic behavior

## Abstract

This research aims to maximize polyethylene terephthalate (PET) nonwoven fabric waste and make it as a new source for benzoic acid extraction using a pyrolysis process. The treatment was performed using a thermogravimetric analyzer (TGA) and released products were characterized using FTIR spectroscopy and gas chromatography–mass spectrometry (GC–MS). The pyrolysis kinetic and thermodynamic behavior of PET fabric was also studied and simulated using different linear and nonlinear models. The results show that the PET fabric is very rich in volatile matter (80 wt.%) and can completely degrade under 490 °C with a weight loss of 84%. Meanwhile, the generated vapor was rich in the carbonylic C=O functional group (FTIR), and the GC–MS analysis concluded that benzoic acid was the major compound with an abundance of 75% that was achieved at the lowest heating rate (5 °C/min). The linear kinetic results showed that PET samples had an activation energy in the ranges of 193–256 kJ/mol (linear models) and ~161 kJ/mol (nonlinear models). The thermodynamic parameters, including enthalpy, Gibbs free energy, and entropy, were estimated in the ranges of 149–250 kJ/mol, 153–232 kJ/mol, and 256–356 J/mol K, respectively. Accordingly, pyrolysis treatment can be used to extract benzoic acid from PET fabric waste with a 134% increase in the benzoic acid abundance that can be recovered from PET bottle plastic waste.

## 1. Introduction

Nonwoven fabrics are a major player in the manufacture of ultrafiltration and nanofiltration membranes that are used as a porous backing to enhance their mechanical performance, durability, and allow them to work in harsh environments and under high pressure [1]. Several types of fabrics have been used for this purpose, such as carbon fiber, glass fiber, polyethylene terephthalate (PET), etc. [2,3,4]. Among the different types of support layers, PET is widely used for this propose due to its high strength, high chemical stability, low production cost, homogeneous porous structure, large pores, high compatibility, strong adhesion to the main filtration film (active layer), and large porosity that allows the target to pass through the membrane [5,6]. PET nonwoven fabric is composed of fibers distributed uniformly manufactured via wet-laying or wet-laid technology [7]. All these distinct characteristics have made it a suitable choice for many separation industries, including water treatment, gas separation, pharmaceuticals, protein extraction, adsorption of Cr(VI), etc. [8,9,10,11,12]. It has also been used with ultrafiltration and nanofiltration membranes, which have high bonding with active layers such as poly(vinylidene fluoride), polysulfone, etc. [3,6]. However, their end-of-life products pose a critical problem that needs to be seriously managed.

In fact, there is no specific technology for the recycling of PET fabrics and they are treated as plastic waste using various mechanical, chemical, and thermal processes [13,14]. Although mechanical treatment (shredding and granulation) accompanied by extrusion processes can be used to convert PET waste into granulated material that can be employed in the production of the secondary products [15], these pellets are usually contaminated with organic or inorganic and heavy metals (having different particle sizes and shapes) remaining from the separation process, which affects the quality, crystallinity, and compatibility of the obtained products [16]. These contaminants can be removed by chemical treatment, including dissolution and a leaching process [17]. However, this practice is undesirable because it consumes a lot of chemicals (solvents and acids) with high toxicity and emissions [18]. For these reasons, the biotechnology on depolymerization of PET and repolymerization of reclaimed monomers from PET is still at lab scale [19]. Therefore, thermal treatments, especially pyrolysis and gasification, are receiving a lot of attention as they are eco-friendly processes characterized by simplicity and low emissions, and do not need any kind of chemical pretreatment [20,21]. The gasification process can convert PET into a syngas product consisting of H_2_ and CO gases. However, this process consumes a lot of energy in the reaction, which takes place at 700 °C [22,23]. In contrast, pyrolysis treatment is performed in an oxygen-free environment with lower temperature, causing the PET molecules to decompose to high-added value chemical compounds, and these contaminations can be consumed as an autocatalyst to increase the abundance of synthesized compounds or remain in the char fraction [24]. 

Pyrolysis has been used to treat end-of-life thin films and their nanocomposite membranes and to study their pyrolysis kinetics [25]. There are several studies in the literature that have been developed to investigate the thermal decomposition of PET based on different viewpoints. For example, Asma Dhahak et al. (2019) studied the slow pyrolysis of PET in the ranges of 410 °C to 480 °C [26]. The results showed that the larger production of benzoic acid compound was produced at 430 °C. Pavel Straka et al. (2021) used slow pyrolysis (400 °C at 25 °C/min) to convert PET into ethylene glycol, benzoic acid, and clean fuel [27]. Yifan Liu et al. (2022) studied the effect of PET plastic bottle sheet size on the energy recovery using pyrolysis treatment [28]. The results showed that a 10 × 10 mm^2^ sheet size and 550 °C are the optimum conditions to achieve the maximum overall energy efficiency. The pyrolysis kinetic behavior of PET has been investigated using different approaches [29,30,31,32,33]; all these studies focused on PET plastic bottles or sheets, and bulk materials or the active layer of the membranes in which the support layer and PET fabric is still missing, as this type of fiber is characterized by a semi-crystalline structure that facilitates its decomposition process [34]. 

In order to encourage the PET fabric producers to invest in this technology, it is necessary to study PET’s pyrolysis properties individually, as single components, to determine its decomposition mechanism, and then study the effect of other additives including the filtration layer, and to avoid the complexity of reactions resulting from including multiple components in the reaction, as PET can usually be found in this type of application as composite waste or mixed with the main separation layer. PET nonwoven fabric is also among the most porous substrates used as a support layer in the production of ultrafiltration and nanofiltration membranes due to its large porosity and high mechanical performance. The huge demand for PET fabrics results in large amounts of waste PET fabrics without specific recycling and management processes. Within this context, this work aims to study the pyrolysis characteristics of PET fabric using a thermogravimetric analyzer (TGA) and its kinetic behavior using linear and nonlinear models, including Flynn–Wall–Ozawa (FWO), Kissinger–Akahira–Sunose (KAS), Friedman, Vyazovkin, and Cai methods [35]. The vapors generated from the thermal decomposition of PET fabric were characterized using Fourier-transform infrared spectroscopy and chromatography–mass spectrometry (GC–MS). 

## 2. Experimental

### 2.1. Materials and PET Feedstock Preparation

**Figure 1:**
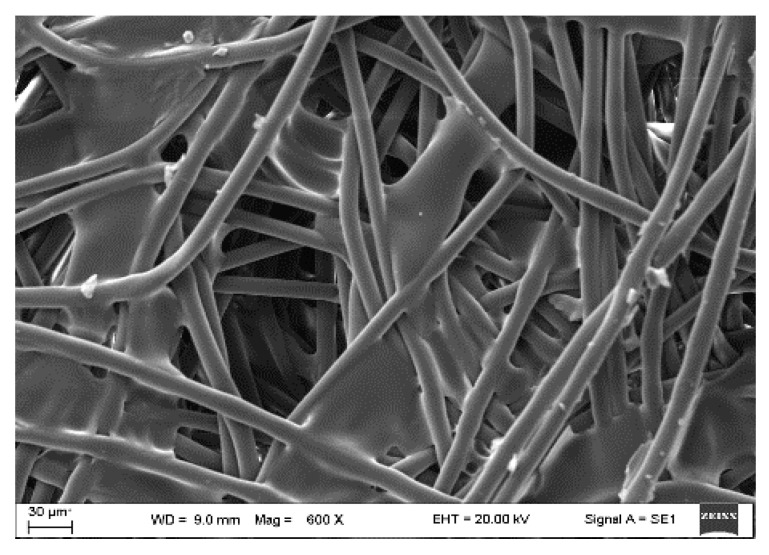
Surface morphology of PET fabric [36].

The nonwoven support fabric polyester layer with thickness of 190 µm (PET; Novatexx 2413) used in the present study was supplied by Freudenberg Group, Weinheim, Germany. A scanning electron microscopy (SEM) was used to observe the surface morphology of the supplied PET feedstock and its SEM image is shown in Figure 1. The measured sample was coated with a very thin gold layer to increase its conductivity and avoid any reflection or deformation of the sample caused by the applied high voltage (20 kV). As shown, the fabric had a complex structure composed of intertwined fibers (in the longitudinal and transverse directions) and loosely arranged in the form of a porous structure [36]. The gases needed for TGA experiments and other analyses were supplied by the Lithuanian Energy Institute, Kaunas, Lithuania. The received PET sheet was crushed into fine particles using an electrical coffee grinder for 10 min, then exposed to a sieving process to obtain uniform PET particles (less than 200 μm) that could be used for thermal experiments using TGA. Finally, the elemental and proximate analysis of the PET fabric was determined based E1756-01, E872-82, and E1755-01 standards [37]. The measurements were made with a Perkin Elmer 2400 CHN analyzer (PerkinElmer, Hongkong, China) and the fixed oxygen and oxygen content was calculated by the difference. The measurements were taken three times to test their repeatability. 

### 2.2. Thermogravimetric Measurements

The thermal degradation profile for the PET fabric was determined using TGA (STA449 F3; NETZSCH, Selb, Germany). The measurements were conducted on 7–11 mg from each membrane batch in nitrogen (60 mL/min) from room temperature to 900 °C. The experiments were performed at various heating rates (5, 10, 15, 20, 25, and 30 °C/min) to study the effect of heating conditions on the degeneration profiles and abundance of the GC–MS compounds. These measurements in various heating conditions are needed in the kinetic molding of the pyrolysis membrane to obtain an accurate result (activation energy). Based on the measured TGA data, DTG data were obtained through the numerical derivation of TGA measurements via Proteus software (https://www.labcenter.com/); then, a DTG profile for each sample was plotted. Based on the measured TGA data and the fitted DTG profiles, the devolatilization index (D_i_) of volatile matters and the heat-resistance index (THRI) parameters were estimated for each batch using Equations (1) and (2) [38].

(1) $$D_{i}=\frac{R_{max}}{T_{i}T_{m}\Delta T}$$

THRI = 0.49 × [T_5_ + 0.6 × (T_30_ − T_5_)] (2)

### 2.3. Chemical Analysis of the Generated Vapor Pyrolysis Compounds

The functional groups of the generated vapor products from decomposition of PET fabric at all the listed heating rates were examined using FTIR coupled with TGA. The chemical compounds of these vapor products were identified and quantified using thermogravimetry–gas chromatography–mass spectrometry (Shimadzu GC-2010, Kyoto, Japan) in an Automation Autoinjector™ unit (ATS Automation, Cambridge, ON, Canada). The GC analysis was conducted in the range of 30–600 m/s within the following conditions: 50 Hz; TCD and injector temperature, 70 and 90 °C, pump and inject time 20 and 30 ms; and column setting, argon, 20 psi, 95 °C, and 110 s) [39].

### 2.4. Pyrolysis Kinetics and Thermodynamic Analysis of PET Membranes

Pyrolysis kinetics were employed to study the activation energy (Ea) needed to decompose each sample individually and its composite, thus determining the degree of complexity of all samples and their energy consumed during the decomposition. This energy was calculated for the whole pyrolysis processes using a Kissinger model (as listed in Equation (3)), and at each conversion rate (y) [40]. There are two common approaches to calculation that can be classified as linear and nonlinear isoconversional models. The linear approaches, including Friedman, Flynn–Wall–Ozawa (FWO), and Kissinger–Akahira–Sunose (KAS) methods, are characterized by simplicity and all their formulas are listed in Equations (2)–(5) [41]. Activation energy can be estimated using the curve fitting approach, which is highly recommended for single reactions, but not multi reactions like in the present case, which investigated composite waste with several subcomponents. In contrast, the linear model, including Vyazovkin and Cai formulas (Equations (2)–(5)), is characterized by its high accuracy and is based on iteration and optimization [42]. Finally, the plotted TGA-DTG profiles of each sample were simulated using the distributed activation energy model (DAEM) and independent parallel reaction kinetic model (IPR) using Equations (6) and (7) [43], followed by determining the deviations (dev.%) between the experimental and simulated profiles using Equation (9). The definitions of all the items used in the presented formulas are shown in Table 1. Finally, the thermodynamic behavior of PET films in terms of enthalpy (ΔH), Gibbs free energy (ΔG), and entropy (ΔS) were studied at 5 and 30 °C/min using Equations (12)–(14). In these formulas, KB, Tm, and h terms are defined as Boltzmann constant (1.3819 × 10^−23^ J/K), maximum decomposition temperature based on DTG curves, and Planck’s constant (6.6269 × 10^34^ J s), respectively [44]. 

## 3. Results and Discussion

### 3.1. Elemental and Proximate Analysis

The elemental and proximate characteristics of the received PET feedstock are listed in Table 2. The elemental analysis showed that carbon (C) was the major element in the sample with content estimated at 62.32 wt.%. This high content of the C element acts as a source of carbon precursor and can be employed to accelerate the conversion rate during the treatment. A small amount of H element (4.2–5 wt.%) was noticed. Meanwhile, the sample did not contain nitrogen (N) and sulfur (S), which reduce the potential for generation of toxic NOx and SO_2_ emissions during industrial scale processing via pyrolysis technology [45]. The proximate analysis showed that PET fabric is very rich in volatile matter (80 wt.%) with a smaller amount (9%) of volatile matter compared to PET plastic waste (88 wt.%) [34]. A high content of fixed carbon (17.89 wt.%) was observed in the tested sample accompanied with a small content of ash (1.9 wt.%). This high content of volatile matter indicates that all samples were rich in hydrocarbons and fixed carbon and ash content, which can help in the conversion process and yield of the recovered chemical compounds [46].

### 3.2. Thermogravimetric Analysis

**Figure 2:**
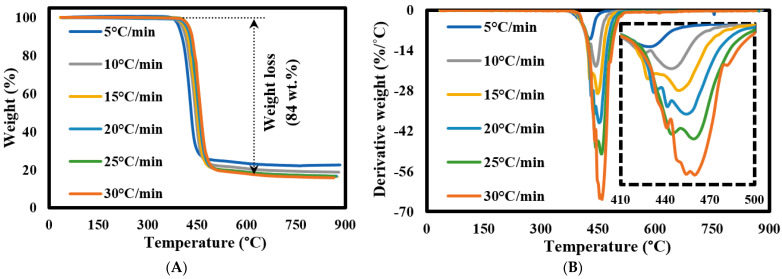
(**A**) TGA and (**B**) DTG curves of PET fabric.

Figure 2 shows the TGA-DTG plots of the PET fabric corresponded to pyrolysis conditions at heating rates of 5, 10, 15, 20, 25, and 30 °C/min. The TGA plots (Figure 2A) showed that the PET substrate had a high thermal stability till 382 °C with almost no weight loss (<0.4 wt.%) in eliminating moisture content in the samples. After that, two thermal decomposition zones were observed till the end of the reaction. The first zone was finished at 490 °C with weight loss of 80 wt.%, while the second degradation zone finished at 770 °C with weight loss < 4 wt.% due to ash formulation. The last degradation region refers to char devolatilization and carbon black formulation. These results match those of the thermal decomposition of PET from waste soft drink bottles [47,48]. Meanwhile, DTG results (Figure 2B) showed that the PET sample decomposed as a single sharp peak, and the intensity of this DTG peak increased with the heating rate rising due to an increase in the generated heat flux that allowed more heat exchange between the surroundings of feedstock and their internal molecules [49]. The porous structure of the tested samples greatly helped to accelerate the exchange decomposition of all organic components involved in the reaction [26]. Finally, all the pyrolysis characteristics of each sample, including melting temperature and glass transition temperature, are illustrated in Table 3. It was noted that the calculated Di value increased when raising the heating rate, which accelerated the volatilization process and inhibited the condensation reaction combined with a greater release of volatiles in the form of an endothermic process via an exothermic process for solid residue formulation [37]. Similarly, the calculated THRI values were increased once heating conditions increased from 201 to 2016 °C due to the higher heating flux and decreasing PET crystallinity [50,51].

### 3.3. Mechanism of PET Membrane Pyrolysis

**Figure 3:**
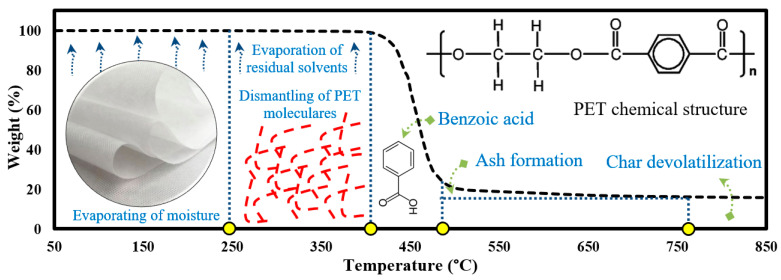
Mechanism of PET fabric pyrolysis.

As illustrated in the above section, the commercial PET fabric decomposed in several sequential stages. This section explains the pyrolysis mechanism of these stages in a simple way based on TGA results that achieved the highest abundance of volatiles (30 °C/min based on GC–MS results). A schematic diagram of the proposed pyrolysis mechanism for PET is shown in Figure 3. As shown, PET offers a high degradation resistance (up to 252 °C) without any weight loss, followed by a very small weight loss (<0.4 wt.%) due to the breakdown of the PET chains into smaller molecules that can easily melt under the applied temperature up to 410 °C [47]. After that, the melt began to decompose quickly (with a decrease of 80 wt.%) into released gaseous hydrocarbons and light hydrocarbons compounds in the form of pyrolysis vapors [48], followed by ash formulation and char devolatilization smoothly and slowly in the following regions till the end of the reaction [49].

As shown, PET fibers had a lower thermal stability compared to virgin PET due to its lower crystallinity and their bigger free space, which allowed the generated heat flux to penetrate this free space and break the Van der Waals bonds of the internal molecules of PET chains combined with several consecutive reactions (random chain scission, side group, and recombination), thus developing smaller molecules rich in gaseous hydrocarbon compounds [51,52]. This structure also led to changes in their chain size and this helped in the digestibility process and converting PET molecules into hydrocarbon and benzoic acid compound [48]. Accordingly, PET texture is a very important factor in improving not only its properties and performance but also its thermal conversion performance and its recyclability, and can help in speeding decomposition and releasing more gaseous hydrocarbons. 

### 3.4. Chemical Analysis of the Synthesized Volatile Products Using TG-FTIR

**Figure 4:**
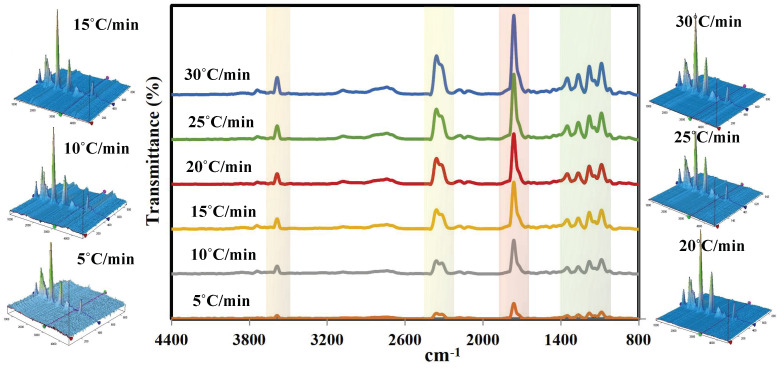
Two- and three-dimensional FTIR spectra of the decomposed PET fabric.

Figure 4 shows TG-FTIR spectra of the generated vapors from the thermal decomposition of PET fabric in the main degradation region at all the specified heating rates (5–30 °C/min). As shown, several function groups were obtained at 1078–1356 cm^−1^ (aromatic C-H bonds; aromatic presence and coordination of benzoyl [53]), 1768 cm^−1^ (carbonylic C=O; showing the presence of aldehydes, ketones, and acids formation), 2265–2393 cm^−1^ (typical place for CO_2_), 2688–2888 cm^−1^ (aromatic C-H bonds (from benzene molecule in PET)), 3581 cm^−1^ (sharp shape of this peak represents acetylene CH≡CR group; the shape is needle-like, very sharp), and 3710 cm^−1^ (hydroxyl OH group; shape of the peak is wide, broad, round) [26]. It was noted that, in all samples, the intensity of these peaks increased gradually when increasing the heating rate from 5 °C/min till 30 °C/min due to increasing the heating flux and cracking rate [49]. Higher heating rates enhanced the decarboxylation reaction and secondary cracking of carbonyl (-C=O-) and carboxyl (-COOH) groups, which contribute in the formation of greater CO_2_ and CO [54].

### 3.5. Chemical Analysis of the Synthesized Chemical Compounds Using GC–MS

**Figure 5:**
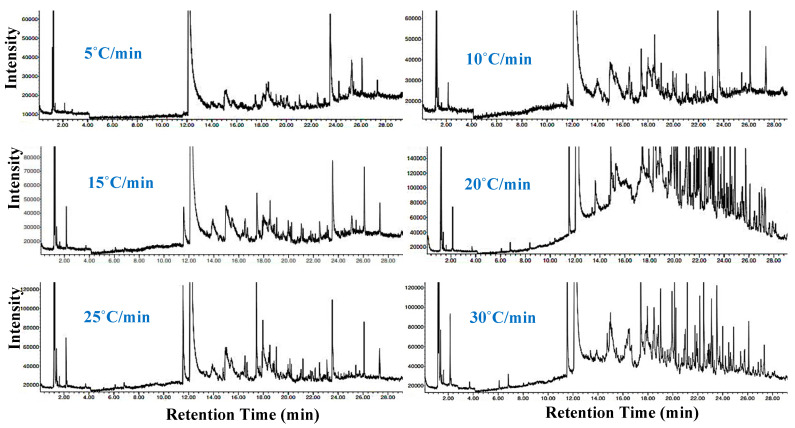
GC–MS analysis of the decomposed PET samples at 5–30 °C/min.

Figure 5 shows GC–MS spectra of the produced volatile species at all heating rates, while the volatile compounds formulated from the pyrolyzed PET samples at the listed heating rates and their intensity are illustrated in Table 4. The GC–MS spectra showed several GC compounds and benzoic acid and acetaldehyde were the major compounds with abundance (peak area) in the ranges of 60.6–74.9% and 6.07–9.89%, respectively. The high intensity of benzoic acid compound was achieved at the lowest heating rate (5 °C/min). This large abundance of benzoic acid in the produced vapor indicates that the PET fabric was converted into high-added value chemical products, which can be used in various applications such as the production of environmental radiocarbon monitoring, phenol, ointments, food, chemicals, pharmaceuticals, etc. [55,56]. Compared to the results of the catalytic pyrolysis of PET waste, which showed that 32 wt.% of the benzoic acid compound could be recovered at a temperature of 450–600 °C [57], the pyrolysis of PET fabric is very promising with a 134% increase in the benzoic acid abundance.

### 3.6. Kinetic and Thermodynamic Analysis of PET Fabric Pyrolysis

#### 3.6.1. Activation Energy for the Entire Thermochemical Process

**Figure 6:**
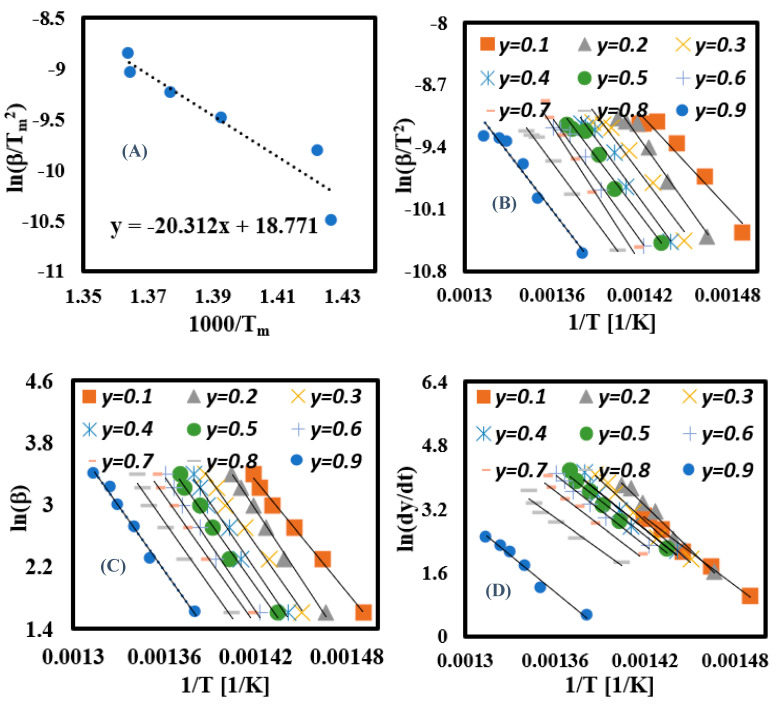
(**A**) Kissinger, (**B**) KAS, (**C**) FWO, and (**D**) Friedman plots of PET samples.

The Kissinger model was used to calculate the Ea of the complete downstream pyrolysis process of PET fabric. The fitting Kissinger relationship is shown in Figure 6A with a slope curve represented by −Ea/(*R* = 8.31 JK^−1^ mol^−1^). Based on the calculation and fitting curves, the entire Ea was estimated at 169 kJ/mol (PET). This value in agreement with the Ea (165.6 kJ/mol) of pyrolysis of PET water bottles, which was calculated based on the ASTM-E698 method [58]. This means the PET geometry does not significantly affect the entire Ea. 

#### 3.6.2. Calculation of Ea for Each Conversion Rate Using Linear Kinetic Methods

**Figure 7:**
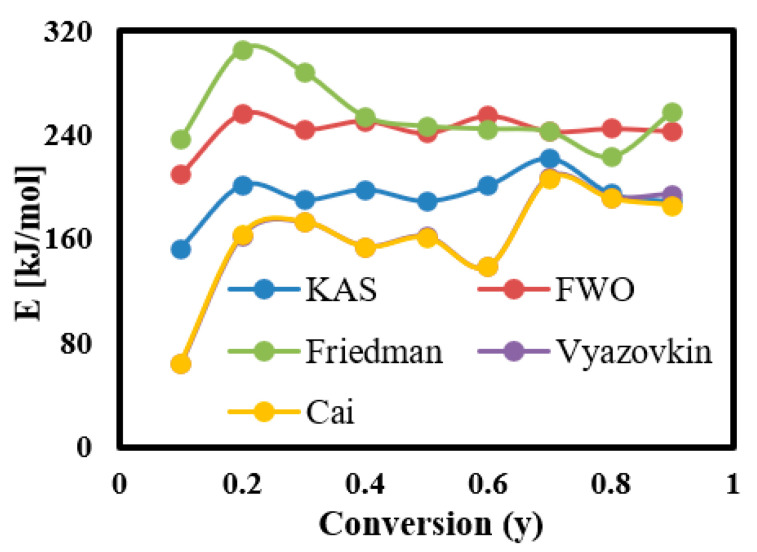
Activation energies–conversion rate curves of PET.

The complexity of pyrolysis of PET fabric in each conversion region in the conversion range of 0.1–0.9 was calculated via linear kinetic models (KAS, FWO, and Friedman). The complexity in terms of Ea was calculated in each model by plotting the curves presented in Figure 6B–D, then obtaining their slopes, which are expressed −Ea/R (KAS and Friedman), −1.0516 Ea/R (FWO). The curves showed nine straight lines representing the Ea at each conversion region; the fitted curves showed random straight lines (at lower and higher conversion regions) due to the occurrence of several reactions at these regions [38], while parallel straight lines were observed in the range of 0.2–0.8. However, in the Friedman model, curves with random distribution were the prevailing lines due to the model’s high sensitivity to noisy data [59,60]. Consequently, in the fitted curves, Ea was calculated and its values as a function of conversion rate (y) are shown in Figure 7. As shown, the Ea value increased as y rose due to involvement of several elements in the thermochemical reaction, thus increasing the reaction’s complexity [43]. Thus, the average Ea was estimated at 193.4 kJ mol^−1^ (KAS), 243.2 kJ mol^−1^ (FWO), and 255.7 kJ mol^−1^ (Friedman), while the coefficient of determination (R^2^) values of each Ea are illustrated in Table 5 with estimated R^2^ values for KAS (0.97), FWO (0.99), and Friedman (0.98). As shown, the KAS and FWO models had higher predictivity compared to Friedman, which means both KAS and FWO linear kinetic methods can be used to investigate the reaction mechanism of PET fabric. These results match the results obtained by Osman et al. (2020) [58].

#### 3.6.3. Calculation of Ea for Each Conversion Rate Using Nonlinear Kinetic Methods

**Figure 8:**
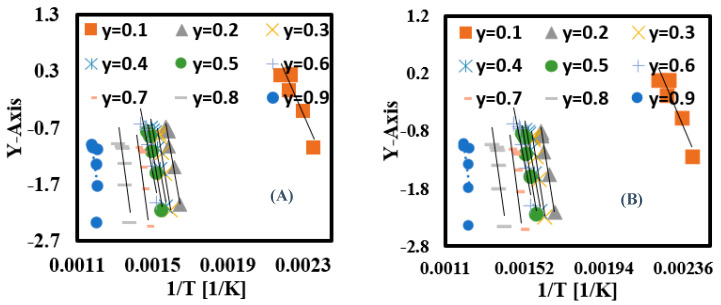
(**A**) Vyazovkin and (**B**) Cai fitting relationships of PET fabric.

The Ea at each conversion zone was recalculated again using the Vyazovkin and Cai numerical integration algorithm nonlinear kinetic methods to obtain accurate Ea values and avoid the noisy results obtained from linear kinetic methods [59,60]. The algorithms were built and solved using Matlab software (https://www.mathworks.com/products/matlab.html) with a 200 kJ/mol as an initial boundary condition followed by running the algorithms several times until the Ea value became constant, thus finding the optimal value of Ea. This goal was achieved with both algorithms after four tries, as illustrated in Table 6. As shown, the Ea became constant after the third try in both algorithms with an estimated average Ea of 161 kJ/mol (Vyazovkin) and 160 kJ/mol (Cai). These values were employed in plotting Vyazovkin and Cai curves (Figure 8), and Y-axes formulas are described in Equations (15) and (16), while R^2^ was estimated as 0.91 for both models, as illustrated in Table 5. As shown, Vyazovkin and Cai algorithms gave similar results and the KAS model produced the values nearest those of other linear model-free models. Their calculated Ea values were quite similar to the Ea values calculated according to the ASTM-E698 method [58]. Therefore, nonlinear algorithms are the most suitable approaches to study the thermal decomposition kinetic properties of PET fabric with high accuracy.

(15) $$\ln\left\{\frac{\beta_{i}}{T_{y,i}^{2}\left[h\left(x_{y,i}\right)-\frac{x_{y,i}^{2}e^{x_{,i}}}{x_{y,i-0.1}^{2}e^{x_{y,i-0.1}}}h\left(x_{y,i-0.1}\right)\right]}\right\}$$

(16) $$\ln\left\{\frac{\beta_{i}}{T_{\alpha ,i}^{2}\left[h\left(x_{\alpha ,i}\right)-\frac{x_{\alpha ,i}^{2}e^{x_{\alpha ,i}}}{x_{\alpha -∆\alpha ,i}^{2}e^{x_{\alpha -∆\alpha ,i}}}h\left(x_{\alpha -∆\alpha ,i}\right)\right]}\right\}$$

#### 3.6.4. Analysis of the Fitted TGA-DTG Curves Using DAEM and IPR

**Figure 9:**
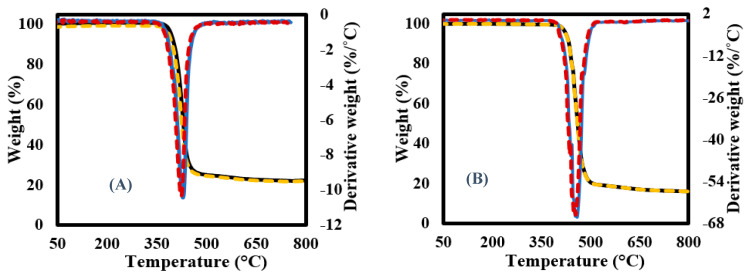
Plots of TGA-DTG curves of PET samples at (**A**) 5 °C/min and (**B**) 30 °C/min.

The experimental data received from TGA-DTG measurements was simulated using DAEM and IPR formulas. The experimental (solid lines) and simulated (dotted lines) TGA-DTG curves at the lowest (5 °C/min) and highest (30 °C/min) heating conditions is shown in Figure 9. As shown, both models succeeded in predicting the thermal decomposition curves of all samples with a close match between the experimental and simulated TGA-DTG curves (a deviation of less than 0.5 using Equation (11)). Finally, the optimum parameters (activation energy “E1 and E2” and pre-exponential “A1 and A2”) used in fitting of these curves are shown in Table 7.

#### 3.6.5. Thermodynamic Analysis

The values of thermodynamic parameters (ΔH, ΔG, and ΔS) calculated based on KAS, FWO, and Friedman results at 5 °C/min and 30 °C/min are shown in Table 8 and Table 9. The calculations showed that these parameters changed with conversion rate changes due to the complex reaction of PET. The parameters were estimated in the ranges of 188–250 kJ/mol (ΔH), 154–232 kJ/mol (ΔG), and 267–356 J/mol K (ΔS) at 5 °C/min; in contrast, these parameters were estimated in the ranges of 188–250 kJ/mol (ΔH), 153–170 kJ/mol (ΔG), and 256–340 J/mol K (ΔS) at 30 °C/min. As shown, the heating rate had a significant effect on the calculated parameters; also, the calculated ΔH parameters had positive values indicating an endothermic nature [61].

## 4. Conclusions

The pyrolysis behavior of polyethylene terephthalate (PET) porous fabric was studied using a thermogravimetric analyzer (TGA), and the formulated vapor pyrolysis products were characterized by TG-FTIR and GC–MS. In addition, the pyrolysis kinetics were studied using linear and nonlinear fitting methods to determine the reactions’ complexity and their activation energies. The TGA results showed that PET fabric decomposed into two stages with a total mass loss of 84% and the main decomposition region was placed in the range of 408–500 °C. The activation energy obtained from linear kinetic models was located in the ranges of 193.4–255.65 kJ/mol with R2 = 0.97–0.99 vs. 160–161 kJ/mol with R2 = 0.91 in case of the nonlinear kinetics. The carbonyl group was the major functional group in the FTIR spectra of the released gases, and the volatile species were rich in benzoic acid compound, comprising 75% of the species, and synthesized at 5 °C/min. The pyrolysis mechanism of PET was also studied to understand the complexity of the reaction that occurred during the thermal degradation process. The results revealed that the radical intermediates play a pivotal role in spreading the pyrolysis chains and in the formed pyrolysis compounds as well. Based on these results, PET waste can be a new source of benzoic acid using the pyrolysis treatment as an eco-friendly emerging technology.

## Figures and Tables

**Table 1:** Formulas used in the modeling of pyrolysis kinetics of PET fabric [42,43,44].

**Equation No.**	**Method**	Expressions
(3)	Kissinger	$\ln\left(\frac{\beta }{T_{m}^{2}}\right)\;\;$=$ln\left(\frac{AR}{E_{a}}\right)\;$ − $\frac{Ea}{RT}$
(4)	KAS	$\ln\left(\frac{\beta }{T^{2}}\right)\;\;$=$ln\left(\frac{AR}{Eag\left(y\right)}\right)\;$ − $\frac{-Ea}{RT}$
(5)	FWO	lnβ = $\left(\frac{lnAEa}{Rgy}\right)\;$ − 5.335 − $\frac{1.0516Ea}{RT}$
(6)	Friedman	$ln\left(\frac{\beta dy}{dT}\right)$ = $ln\left(Af\left(y\right)\right)\left(\frac{-Ea}{RT}\right)$
(7)	Vyazovkin	$\left(\alpha \right)=\int_{0}^{\alpha }\frac{dy}{f(y)}=A\int_{0}^{t}exp(-E/RT)dt$
(8)	Cai	$\ln\left\{\frac{\beta_{i}}{T_{y,i}^{2}\left[h\left(x_{y,i}\right)-\frac{x_{y,i}^{2}e^{x_{y,i}}}{x_{y-∆y,i}^{2}e^{x_{y-}∆y,i}}h\left(x_{y-∆y,i}\right)\right]}\right\}=\ln\left\{\frac{A_{y-∆y/2}R}{E_{y-∆y/2}g(y,y-∆y)}\right\}-\frac{E_{y-∆y/2}}{RT_{y,i}}$
(9)	DAEM	$\ln\left(\frac{\beta }{T^{2}}\right)\;\;$=$ln\left(\frac{AR}{Ea}\right)+0.6075$ − $\frac{Ea}{RT}$
(10)	IPR	${\frac{dm}{dt}}^{calc}=-(m_{0}-m)\sum_{i=1}^{3}\cdot C_{i}\frac{dX_{i}}{dt}$
(11)	Dev.%	Dev.(%) = $\frac{100\sqrt{{F.O.}_{DTG}(Z-N)}}{max(\left|dm/dt\right|)}$
(12)	ΔH	Δ*H* = *Ea* − *RT_m_*
(13)	ΔG	ΔG = Ea + *RT_m_* $\ln\left(\frac{K_{B}T_{m}}{hA}\right)\;\;$
(14)	ΔS	ΔS = $\frac{\Delta H-\Delta G}{T_{m}}$

**Table 2:** Elemental and proximate analysis of PET samples.

Elemental Analysis (wt.%)		Proximate Analysis (wt.%)	
Nitrogen (N)	<0.01	Moisture	0.27 ± 0.04
Carbon (C)	62.318 ± 0.035	Volatiles	79.92 ± 0.28
Hydrogen (H)	4.221 ± 0.024	Fixed Carbon	17.89 ± 0.12
Sulfur (S)	<0.01	Ash	1.92 ± 0.01
Oxygen (O)	33.46 ± 0.021		

**Table 3:** The pyrolysis characteristics of the decomposed PET samples.

Pyrolysis Parameters	5 °C/min	10 °C/min	15 °C/min	20 °C/min	25 °C/min	30 °C/min
Ti (°C)	362	367	375	385	394	396
Tm (°C)	429	445	451	453	456	460
Tf (°C)	484	498	510	516	519	521
Rmax (%/min)	10	19.4	28.9	39.2	50	65.9
Di (% min^−1^ °C^−3^)	1.95 × 10^−6^	3.05 × 10^−6^	4.27 × 10^−6^	5.35 × 10^−6^	6.63 × 10^−6^	8.82 × 10^−6^
ΔT	33	39	40	42	42	41
T5	393.3	413.2	404.9	416.5	423.7	424.7
T30	420.9	436.6	433.5	444.1	446.7	450.5
THRI	200.8	209.3	206.8	212.2	214.4	215.7

**Table 4:** GC–MS compounds generated from the pyrolysis of PET fabric.

**5 °C/min**			10 °C/min			15 °C/min			20 °C/min			25 °C/min			30 °C/min		
Time (min.)	GC Compounds	Area (%)	Time (min.)	GC Compounds	Area (%)	Time (min.)	GC Compounds	Area (%)	Time (min.)	GC Compounds	Area (%)	Time (min.)	GC Compounds	Area (%)	Time (min.)	GC Compounds	Area (%)
1.181	Carbon dioxide	2.12	1.187	Carbon dioxide	2.17	1.187	Carbon dioxide	2.59	1.181	Carbon dioxide	1.90	1.194	Carbon dioxide	3.21	1.187	Carbon dioxide	3.66
1.239	Acetaldehyde	7.40	1.245	Acetaldehyde	8.46	1.239	Acetaldehyde	9.46	1.239	Acetaldehyde	6.07	1.246	Acetaldehyde	9.89	1.239	Acetaldehyde	9.60
12.177	Benzoic acid	74.90	11.628	N-[5-(m-Tolyl)-1,3,4-thiadiazol-2-1326yl]benzimidic acid	1.02	2.158	Benzene	0.67	11.531	Vinyl benzoate	2.70	2.164	Benzene	0.81	2.151	Benzene	0.85
15.030	2-n-Propoxyamphetamine	1.32	12.190	Benzoic acid	71.82	11.602	Benzenebutanoic acid, .gamma.-oxo-	1.84	12.197	Benzoic acid	60.60	11.602	Vinyl benzoate	4.40	11.556	Vinyl benzoate	5.63
17.514	2,4-Dimethylamphetamine	0.60	14.965	2-n-Propoxyamphetamine	0.98	12.203	Benzoic acid	73.40	14.894	4-Ethylbenzoic acid	2.28	12.223	Benzoic acid	68.88	12.216	Benzoic acid	62.53
19.176	Acetic acid, 2-(1-methyl-2-oxohydrazino)-, N′-[(E)-(2-hydroxyphenyl) methylidene]hydrazide, N-oxide	0.39	17.488	(3,4,5,6-Tetrahydro-2H-[2,3′]bipyridinyl-1-yl)acetic acid hydrazide	1.79	14.965	4-Ethylbenzoic acid	1.26	17.424	4-Methyl-2,6-dihydroxyquinoline	1.66	14.959	4-Ethylbenzoic acid	1.60	17.449	2-t-Butyl-1-methyl-3-phenyl-imidazolidin-4-one	4.18
21.046	2-Fluorenamine	0.60	18.523	Phthalic acid, 2-hexyl ester	0.93	17.482	2-t-Butyl-1-methyl-3-phenyl-imidazolidin-4-one	1.90	21.150	Octadecanal	3.72	17.456	Hydantoin, 5-ethyl-5-phenyl-, (.+/-.)-	3.99	19.028	Cyclododecene, 1-methyl-	1.93
22.527	N-Dodecylmethylamine	1.20	21.039	9-Methyl-9H-carbazole	0.91	18.530	Terephthalic monohydroxamic acid	0.72	21.777	Cyclononasiloxane, octadecamethyl-	2.05	17.986	Terephthalic monohydroxamic acid	2.39	20.127	16-Octadecenal	1.58
23.581	Benzoic acid, 2-propenyl ester	9.48	22.514	L-Alanine-4-nitroanilide	0.96	19.054	Benzyl alcohol, .alpha.-(1-aminoethyl)-m-hydroxy-, (-)-	0.42	22.152	Oxirane, heptadecyl-	2.45	23.556	Benzamide, N-hydroxy-N-(2-hydroxy-3-phenoxypropyl)-	2.81	20.237	1,1,1,5,7,7,7-Heptamethyl-3,3-bis(trimethylsiloxy)tetrasiloxane	0.85
24.222	Phenol, 2-[(N,N-dimethylamino)methyl]-3,5-dimethyl-	0.40	23.569	Benzoic acid, 3,3,5-trimethyl-6-oxo-2-phenyl-3,6-dihydro-2H-pyran-4-yl ester	7.35	19.992	Phosphinic fluoride, diphenyl-	0.39	22.469	4,4′-(Hexafluoroisopropylidene)diphenol	8.48	26.092	Benzonitrile, m-phenethyl-	1.29	21.169	Hexadecanal	1.67
26.085	2-Amino-1-(o-methoxyphenyl)propane	1.60	26.091	Azetidine, 1-benzyl-3,3-dimethyl-2-phenyl-	2.38	21.046	Dimethyl-(2-thioxo-[1,2,3]dioxaphosphinan-2-yl)-amine	0.55	23.097	Oleyl alcohol, methyl ether	2.26	27.340	Tetrasiloxane, decamethyl-	0.74	22.159	Hexadecanal	0.97
			27.340	1-Propene, 3-(2-cyclopentenyl)-2-methyl-1,1-diphenyl-	1.23	22.527	1-Methyl-2-phenoxyethylamine	0.77	23.194	Cyclodecasiloxane, eicosamethyl-	1.71				22.495	4,4′-(Hexafluoroisopropylidene)diphenol	2.59
						23.575	Butanedioic acid, 2,3-bis(benzoyloxy)-, [S-(R*,R*)]-	3.68	24.009	Oxirane, hexadecyl-	1.44				23.109	Oxirane, heptadecyl-	1.16
						26.091	Benzonitrile, m-phenethyl-	1.60	24.481	Hexasiloxane, tetradecamethyl-	1.64				23.543	1,2-Ethanediol, dibenzoate	1.78
						27.340	1H-Indole, 5-methyl-2-phenyl-	0.75	25.729	Cyclononasiloxane, octadecamethyl-	1.05				26.091	Azetidine, 1-benzyl-3,3-dimethyl-2-phenyl-	1.00

**Table 5:** The estimated activation energy of decomposed PET fabric at different conversion rates.

y	KAS			FWO			Friedman			Vyazovkin		Cai	
	Ea (KJ/mol)	R^2^	A (1/s)	Ea (KJ/mol)	R^2^	A (1/s)	Ea (KJ/mol)	R^2^	A (1/s)	Ea (kJ/mol)	R^2^	Ea (KJ/mol)	R^2^
0.1	153.19	0.95	8 × 10^8^	209.51	1.00	1.65 × 10^18^	237.09	0.99	7.63 × 10^20^	64.35	0.89	64.83	0.86
0.2	201.87	0.97	4 × 10^11^	256.76	1.00	6.03 × 10^20^	305.76	1.00	3.23 × 10^25^	162.31	0.98	163.88	0.98
0.3	190.31	0.96	2 × 10^10^	244.48	1.00	2.35 × 10^19^	288.20	0.99	4.81 × 10^23^	172.97	0.99	173.58	0.99
0.4	198.31	0.98	3 × 10^10^	250.98	0.99	2.72 × 10^19^	254.06	0.96	4.72 × 10^21^	154.16	0.97	153.98	0.97
0.5	189.29	0.98	3 × 10^9^	241.40	0.99	3 × 10^18^	246.86	0.98	1.03 × 10^20^	161.89	0.97	161.32	0.97
0.6	201.33	0.98	1 × 10^10^	254.89	0.99	1.34 × 10^19^	244.86	0.99	3.98 × 10^19^	138.67	0.89	138.57	0.87
0.7	222.50	0.98	2 × 10^11^	242.81	0.99	1.08 × 10^18^	242.71	0.98	1.39 × 10^19^	207.41	0.92	206.92	0.91
0.8	195.03	1.00	2 × 10^9^	244.97	0.98	8.94 × 10^17^	223.43	0.95	2.27 × 10^17^	191.77	0.82	191.29	0.81
0.9	188.80	0.96	3 × 10^8^	242.84	0.99	2.7 × 10^17^	257.90	0.98	8.42 × 10^18^	194.42	0.80	186.18	0.81
Avg.	193.40	0.97	8 × 10^10^	243.18	0.99	7.49 × 10^19^	255.65	0.98	3.64 × 10^24^	160.88	0.91	160.06	0.91

**Table 6:** The determined activation energy at different number of iterations using Vyazovkin and Cai models.

y	The Activation Energy (kJ/mol)					The Activation Energy (kJ/mol)			
	Vyazovkin					Cai			
	Initial Value	First Iteration	Second Iteration	Third Iteration	Fourth Iteration	First Iteration	Second Iteration	Third Iteration	Fourth Iteration
**0.1**	200	62.24	62.19	64.35	64.35	64.93	64.35	64.83	64.83
**0.2**	200	156.91	156.39	162.31	162.31	162.36	161.82	163.88	163.88
**0.3**	200	166.59	166.70	172.97	172.97	172.37	172.48	173.58	173.58
**0.4**	200	149.04	148.72	154.16	154.16	154.21	153.88	153.98	153.98
**0.5**	200	156.50	156.13	161.89	161.89	161.93	161.55	161.32	161.32
**0.6**	200	133.93	133.84	138.67	138.67	138.58	138.48	138.57	138.57
**0.7**	200	200.40	199.73	207.41	207.41	207.35	206.66	206.92	206.92
**0.8**	200	185.36	184.87	191.77	191.77	191.79	191.29	191.29	191.29
**0.9**	200	179.83	179.35	194.42	194.42	186.07	185.57	186.18	186.18
**Avg.**	200	154.53	154.21	160.88	160.88	159.95	159.56	160.06	160.06

**Table 7:** The optimum parameters used in DAEM and IPR models.

Model	DAEM	IPR
E1	243.15	222.17
A1	4.20 × 10^16^	1.66 × 10^12^
E2	301.51	284.76
A2	4.62 × 10^16^	1.77 × 10^13^

**Table 8:** Thermodynamic parameters for PET pyrolysis at 5 °C/min.

y	*KAS*			*FWO*			*Friedman*		
	ΔH (kJ/mol)	ΔG (kJ/mol)	ΔS (J/mol K)	ΔH (kJ/mol)	ΔG (kJ/mol)	ΔS (J/mol K)	ΔH (kJ/mol)	ΔG (kJ/mol)	ΔS (J/mol K)
0.1	151	176	214.7	207	183	294.9	231	133	329.2
0.2	196	223	278.9	251	154	357.2	300	140	427.0
0.3	184	229	262.5	239	161	339.7	282	147	402.0
0.4	192	234	273.8	245	167	349.0	248	140	353.4
0.5	183	239	261.0	236	170	335.3	241	155	343.1
0.6	195	244	278.1	249	175	354.5	239	158	340.3
0.7	217	248	308.3	237	177	337.3	237	162	337.2
0.8	189	247	269.2	239	181	340.4	218	167	309.7
0.9	183	252	260.3	237	186	337.3	252	180	358.8
Avg.	188	232	267.4	238	173	338.4	250	154	355.6

**Table 9:** Thermodynamic parameters for PET pyrolysis at 30 °C/min.

y	*KAS*			*FWO*			*Friedman*		
	ΔH (kJ/mol)	ΔG (kJ/mol)	ΔS (J/mol K)	ΔH (kJ/mol)	ΔG (kJ/mol)	ΔS (J/mol K)	ΔH (kJ/mol)	ΔG (kJ/mol)	ΔS (J/mol K)
0.1	151	130	205.6	207	182	282.3	231	129	315.0
0.2	196	180	266.8	251	150	341.8	300	133	408.6
0.3	184	150	251.1	238	158	325.0	282	141	384.7
0.4	192	160	262.0	245	163	333.9	248	135	338.1
0.5	183	137	249.7	235	167	320.8	241	151	328.3
0.6	195	157	266.1	249	171	339.2	239	155	325.5
0.7	216	196	295.0	237	175	322.7	237	159	322.6
0.8	189	141	257.6	239	178	325.6	217	165	296.3
0.9	183	123	249.1	237	183	322.7	252	177	343.3
Avg.	188	153	255.9	238	170	323.8	250	149	340.3
